# Clear Cell Sarcoma of the Kidney: Report of Two Cases

**Published:** 2014-09-01

**Authors:** Shalini Sinha, Nita Khurana, Yogesh Kumar Sarin

**Affiliations:** 1 Department of Pediatric Surgery, Maulana Azad Medical College, New Delhi; 2 Department of Pathology, Maulana Azad Medical College, New Delhi

**Keywords:** Clear cell sarcoma, Kidney, Wilm's tumor

## Abstract

Clear cell sarcoma of kidney (CCSK) is an aggressive renal neoplasm. We report two boys aged three and half, and three years with CCSK, one of whom had a disease free survival of four years and eight months. These patients were managed with surgery, chemotherapy and radiotherapy. One of the patients discontinued treatment early and lost to follow up. Aggressive multimodality therapy is the keystone to improved outcome.

## INTRODUCTION

CCSK or bone metastasizing renal tumor of childhood is now recognized as a unique pediatric renal neoplasm with distinctive histologic and clinical features.[1] This aggressive tumor is no longer classified with Wilms’ tumor (WT) and comprises approximately 4-6% of all pediatric renal tumors.[2] It is a highly malignant neoplasm with a greater relapse rate than WT and late recurrence.[3,4] Metastases from CCSK occurs not only to the lymph nodes and lungs as in WT but also to bone, liver and brain.[2,5] It is resistant to conventional therapy, but often responsive to doxorubicin containing regimens.

We report 2 cases of CCSK.

## CASE REPORT

**Case 1**

A 3½ -year-old boy presented with a painful lump in his right abdomen for one week. He had a large, hard, irregular right renal mass measuring 12cms x 10cms, which was crossing the midline. An ultrasonogram done elsewhere was reported as liver abscess. Repeat sonography and CECT abdomen showed a 10 cms x 8.5 cms x 7.6 cms heterogenous mass arising from and almost replacing the right kidney. It was abutting the inferior surface of the liver superiorly and had necrotic areas within. In its infero-lateral part, the mass also abutted the anterior abdominal wall and the inferior vena cava (IVC) was attenuated at the same level (Fig. 1). Findings were consistent with WT and there were no lung metastasis on chest x-ray.

**Figure:**
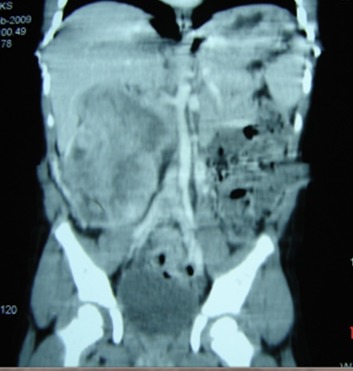
Figure 1:CECT abdomen in case 1 showing the large right renal tumour with necrotic areas, abutting the liver.

A trucut biopsy was sent and while awaiting the histopathology (HPE) report which took unusually long, he received two weeks of chemotherapy with Vincristine and Actinomycin D without any response. The trucut biopsy confirmed CCSK with immunohistochemistry (IHC) positive only for vimentin. Bone scan and CT Brain were normal. Complete excision of the tumor with lymph node sampling was done and final HPE was consistent with CCSK with positive ipsilateral hilar lymph nodes (Stage 3); IHC was positive for vimentin (Fig. 2, 3). He received local radiation therapy (10.8 Gy in 6 fractions) and adjuvant chemotherapy for 24 weeks with vincristine, doxorubicin, cyclophosphamide, and etoposide. He developed varicella infection after week 4 chemotherapy (treated with 1500 mg/m2/day intravenous acyclovir in 3 divided doses for 10 days). Ten days after completion of chemotherapy (i.e. 5 months after the initial varicella infection) he developed Herpes Zoster which was confirmed by Tzanck test. He was treated with oral acyclovir (80 mg/kg/day in 4 divided doses for 5 days). Two months after completion of therapy he presented with headache, fever and severe anaemia (Hb 2.2 G/dl). Imaging did not show any recurrence or metastasis. Bone marrow aspiration revealed megaloblastic anemia which was treated with vitamin B12 and folic acid. Thereafter, he has had no further problems and was kept on a close follow-up every 3 months with clinical examination and ultrasonography of the abdomen. Yearly CECT of abdomen and CT of brain were done. He has a disease free survival (DFS) of 4 years and 8 months till date. 

**Figure:**
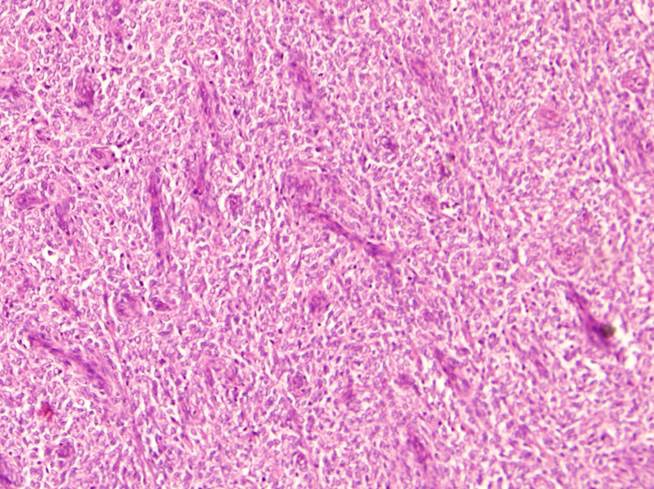
Figure 2:H and E slide showing cellular tumour composed of small ovoid cells with clear cytoplasm with prominent vascularity.

**Figure:**
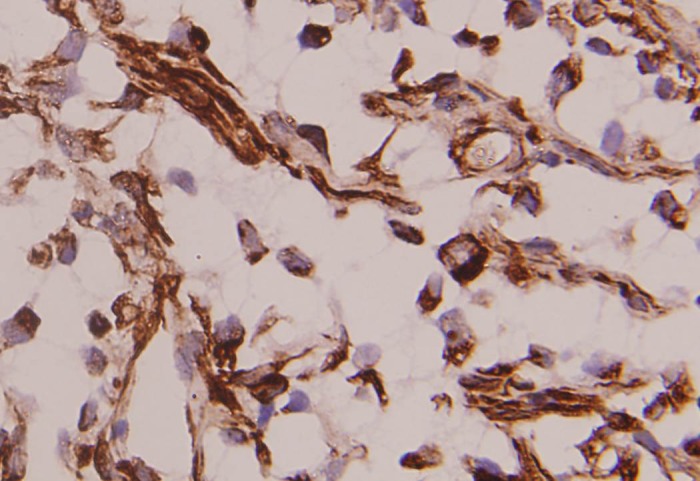
Figure 3:IHC showing positive reaction for Vimentin

**Case 2**

A 3-year-old boy presented with a large lump in the right flank of 15 days duration without any constitutional symptoms. On examination, there was a 12 cms x 10 cms firm mass in the region of the right kidney reaching up-to the midline. He was anemic and hypoalbuminemic. Metastatic workup was negative. Ultrasonography done elsewhere was reported as WT. Repeat imaging including CECT abdomen confirmed a 12 cms x 9 cms x 9 cms heterogenous mass replacing almost the entire right kidney with few cystic areas within. The opposite kidney was normal and IVC was spared. Trucut biopsy revealed CCSK. He was taken up for upfront surgery and complete excision along with lymph node sampling was done. The tumour was abutting the IVC, without any infiltration, and was carefully separated from it. HPE was consistent with CCSK stage I. He was planned for postoperative chemotherapy and radiotherapy. However the patient had early abandonment of therapy after a single cycle of chemotherapy with vincristine, doxorubicin, cyclophosphamide, and etoposide. Several attempts to contact the patient’s family failed. The reasons for abandonment of therapy are yet unknown to us.

## DISCUSSION

Although this highly malignant neoplasm occurs in the same age range as WT, there are no specific radiological features to help distinguish CCSK from WT.[3] Argani et al have described histopathology of CCSK in detail.[6] Gross findings include large tumour size (>10 cms in diameter), mucoid texture, foci of necrosis, and prominent cyst formation. They described nine different histologic patterns (classic, myxoid, sclerosing, cellular, epithelioid, palisading, spindle, storiform, and anaplastic); most tumours containing a mixture of these.[6] Differential diagnoses for CCSK on HPE include blastemal WT and primitive neuroectodermal tumor, both of which have prominent vascular patterns. Reactivity for Vimentin seen on IHC is another helpful tool in excluding other paediatric renal neoplasms.[2] As was seen in both our patients, IHC made it possible to accurately diagnose this rare tumour on pre-treatment trucut biopsy.

The current Children's Oncology Group protocol (AREN0321), follows the NWTS-5 regime for all CSSK patients (nephrectomy followed by radiotherapy and chemotherapy with cyclophosphamide, etoposide, vincristine, and doxorubicin for 24 weeks). Exceptions are those with stage IV disease who receive upfront therapy with irinotecan and vincristine; and those with stage I disease and negative lymph node sampling who do not undergo radiation therapy to the tumor bed. Four important poor prognostic factors identified by NWTS studies are treatment with doxorubicin, beyond stage I, age at diagnosis >2 years and tumour necrosis.[3,6] Reported overall survival (OS) is 69%; stage I OS is 98%. However relapse rates are high and often occur late (30% occurring after 2 years).[7]

Data analysed from the SIOP 93-01/2001 trials show 5 year event free survival (EFS) and OS as 79% and 86% for CSSK.[7] Adverse prognostic factors identified were young age and stage IV disease.

The co-morbidity of Varicella zoster in case 1 is known to occur in immunocompromized previously unimmunized children undergoing chemotherapy and can be potentially fatal.

To summarize, it is possible to diagnose CCSK on trucut biopsy. Lack of response to preoperative chemotherapy for WT should raise suspicion of non-Wilms’ renal tumours. Aggressive multimodality therapy is the keystone to improved outcome.

## Footnotes

**Source of Support:** Nil

**Conflict of Interest:** None declared

